# Optimizing Anticoagulation in Valvular Heart Disease: Navigating NOACs and VKAs

**DOI:** 10.3390/jpm14091002

**Published:** 2024-09-20

**Authors:** Anca Ouatu, Oana Nicoleta Buliga-Finiș, Daniela Maria Tanase, Minerva Codruta Badescu, Nicoleta Dima, Mariana Floria, Diana Popescu, Patricia Richter, Ciprian Rezus

**Affiliations:** 1 Faculty of General Medicine, “Grigore T. Popa” University of Medicine and Pharmacy, 16 University Street, 700115 Iasi, Romania; anca.ouatu@umfiasi.ro (A.O.); daniela.tanase@umfiasi.ro (D.M.T.); nicoleta.dima@umfiasi.ro (N.D.); mariana.floria@umfiasi.ro (M.F.); diana.popescu@umfiasi.ro (D.P.); patricia.richter@umfiasi.ro (P.R.); ciprian.rezus@umfiasi.ro (C.R.); 2 Department of Internal Medicine, IIIrd Medical Clinic, “Sf. Spiridon” Emergency Hospital, 1 Independentei Street, 700111 Iasi, Romania

**Keywords:** valvular heart disease, NOACs, VKA, mitral stenosis, aortic stenosis, TAVI

## Abstract

Background/Objectives: Non-vitamin K antagonist oral anticoagulants (NOACs) have demonstrated similar effectiveness and safety profiles to vitamin K antagonists (VKAs) in treating nonvalvular atrial fibrillation (AF). Given their favorable pharmacological profile, including the rapid onset and offset of action, fixed dosing, and predictable pharmacokinetics with a consistent dose-response relationship, reducing the need for frequent blood tests, researchers have investigated the potential of NOACs in patients with AF and valvular heart disease (VHD). Methods: Clinical trials, excluding patients with mechanical prosthetic valves or moderate/severe mitral stenosis, have shown the benefits of NOACs over VKAs in this population. However, there is a need for further research to determine if these findings apply to mechanical valve prostheses and NOACs. Results: Several ongoing randomized controlled trials are underway to provide more definitive evidence regarding NOAC treatment in moderate to severe rheumatic mitral stenosis. Importantly, recent trials that included patients with atrial fibrillation and bioprosthetic valves (also transcatheter heart valves) have provided evidence supporting the safety of NOACs in this specific patient population. Ongoing research aims to clearly define the specific scenarios where NOACs can be safely and effectively prescribed for various types of VHD, including moderate/severe mitral stenosis and mechanical valves. Conclusions: The aim of this review is to accurately identify the specific situations in which NOACs can be prescribed in patients with VHD, with a focus centered on each type of valvulopathy.

## 1. Introduction

The prevalence of VHD is increasing in the elderly population above 75 years of age [1]. Many patients are likely to have underlying native VHD in addition to AF as VHD becomes more common in the general population and as they age [2]. The most common cause of valve disease in the world is rheumatic heart disease (RHD), which affects 41 million individuals worldwide, or around 0.4% of the total population, mostly in low- and middle-income nations [3,4,5]. Atrial fibrillation (AF) can occur in people with RHD as a result of elevated atrial pressure, persistent inflammation, fibrosis, and left atrial enlargement. Recently, a study involving 75,637 RHD participants across 42 countries found that the average prevalence of AF was 32.9%, with the highest frequency reaching approximately 80% [6].

In the first comprehensive investigation of AF and VHD using electronic health records (EHR), certain VHD subtypes showed low utilization of oral anticoagulation (OAC) despite a significant prevalence and an elevated risk of adverse events [7]. The prevalence of VHD was significant (11.3% at baseline) among 76,019 AF cases, and it grew over time (from 11.4% in 1998 to 17.6% in 2010). The highest thromboembolic risk was associated with the next VHD subcategories: AF with a mechanical valve, mitral stenosis, and aortic stenosisthe poorest results in aortic stenosis [7].

Non-vitamin K antagonists oral anticoagulants (NOACs) are commonly utilized for the treatment of nonvalvular AF both safely and are effectively comparable with vitamin K antagonists (VKA). NOACs and VKA have been compared in randomized trials investigating stroke prevention in patients with nonvalvular AF. The first results showed that NOACs were equally safe and effective as VKA [8,9,10,11]. The most recent guidelines for the diagnosis and management of AF recommend NOACs as an initial treatment option to prevent thrombo-embolic events in nonvalvular AF due to the multiple benefits when compared to VKA [12]. With the predictable pharmacokinetics of NOAC, there is no need for routine monitoring (unlike warfarin, which requires regular INR (International Normalized Ratio) monitoring), there are fewer drug and food interactions, particularly with vitamin K-rich foods and other drugs (certain antibiotics, antifungals, nonsteroidal anti-inflammatory drugs, and corticosteroids increase VKA effects when associated), rapid onset (within a few hours), an offset of action, and standardized dosage with no need for frequent adjustments contribute to an improved adherence among patients, which in turn can improve clinical outcomes.

Despite their initial approval exclusively for ‘non-valvular AF’, NOACs have emerged as a viable choice for an expanding cohort of patients with VHD. Since NOACs have proven that they are not inferior to warfarin in preventing strokes with less severe bleeding in individuals with non-valvular AF, they have become a popular substitute for VKAs [13]. The main studies comparing NOACs with VKAs showed that the impacts of NOACs on stroke/systemic embolism and bleeding risk in patients with VHD, with the exception of moderate/severe mitral stenosis and mechanical prosthetic heart valves, were in agreement with the findings of the primary trials [14].

In reality, the term “non-valvular” is often misunderstood as excluding all types of valvular heart diseases, which is a misconception. The term “non-valvular” actually specifically relates to AF that occurs without the presence of a few categories of valvular conditions, such as significant mitral stenosis or mechanical heart valves [12]. Many patients with atrial fibrillation have shown evidence of valvular involvement, highlighting the need to clarify the term “non-valvular” to ensure that NOACs are not inappropriately withheld due to the mistaken belief that these patients have valvular atrial fibrillation. A new agreement document has presented the EHRA (Evaluating Heart Valve, Rheumatic, or Artificial) classification system in order to direct the choice of oral anticoagulants for the AF and VHD populations [15]. EHRA type 1 VHD refers to patients with moderate to severe mitral stenosis of rheumatic origin or mechanical heart valves, and these individuals are recommended to receive vitamin K antagonists (VKAs) [16]. Studies have indicated that patients with AF and type 1 VHD possess a considerable high risk of thromboembolic events, providing a 25% yearly incidence rate without anticoagulation, reduced to 0.8% with anticoagulation therapy [17]. On the other hand, type 2 VHD incorporates conditions such as mitral regurgitation, aortic regurgitation, aortic stenosis, tricuspid regurgitation, and in patients with bioprosthetic valves or valve repairs. In patients diagnosed with atrial fibrillation and type 2 valvular heart disease, treatment decisions regarding the use of vitamin K antagonists or non-vitamin K antagonist oral anticoagulants are guided by the patient’s CHA2DS2-VASc score [15].

Cardiologists in practice, however, found this language confounding. Only 57% of cardiologists and internists surveyed between 2011 and 2012 felt that the current definitions of non-valvular AF were adequately precise [18]. In a survey assessing the classification of various situations as valvular AF, only 30% of respondents correctly identified that atrial fibrillation associated with valvular conditions, excluding those related to the mitral valve, should be categorized as non-valvular. Additionally, 24% knew that AF with mitral regurgitation should be considered non-valvular, 29% recognized that AF with an aortic bioprosthesis is classified as non-valvular, and just 13% understood that the severity of mitral valve stenosis (moderate-severe vs. mild) is crucial in order to differentiate between valvular and non-valvular AF [19].

The absence of reliable data from major clinical trials is the reason why international guidelines recommend NOACs as a class III recommendation for patients with AF and moderate or severe mitral stenosis. The purpose of this review is to accurately identify the specific situations in which NOACs can be prescribed in patients with VHD, with a focus centered on mitral and aortic valvulopathy. We clarify misconceptions regarding the terms “valvular” and “non-valvular” AF and assess the current proof on the safety and effectiveness of NOACs in patients with VHD other than moderate to severe mitral stenosis and mechanical mitral valves, but also spotlight ongoing research that seeks to explore the possible use of NOACs in VHD contexts that have not been covered in the major NOAC trials.

Regardless of the underlying cardiac rhythm, an elevated risk of ischemic episodes is linked to every valve dysfunction, with mitral stenosis (MS) being the most prevalent type of valve stenosis [20]. Patients who have rheumatic mitral stenosis are significantly more likely to experience thromboembolism when they have AF [21]. Patients with AF who have mechanical heart valve disease or moderate to severe mitral stenosis should take warfarin or other vitamin K antagonists for oral anticoagulation, since there are not enough trials assessing the safety and efficacy of NOACs versus VKAs. These two conditions have been typically left out of randomized controlled trials (RCTs) that compared warfarin to NOACs in patients with nonvalvular AF [8,9,10,11,22]. Additionally, for individuals with mechanical mitral valves, dabigatran raises the possibility of severe bleeding and thromboembolic complications compared to VKAs, making NOACs contraindicated in these cases [23]. Nevertheless, the phrase “nonvalvular AF” is misleading because the previously stated trials involved individuals with various native valve diseases. When prescribing NOACs to AF patients with any type of VHD, some doctors may hesitate due to the term “nonvalvular heart disease”, which was utilized in the initial NOAC studies. However, current guidelines (2021 ESC Guidelines for the Diagnosis and Management of AF and 2023 ACC/AHA/ACCP/HRS Guideline for the Diagnosis and Management of Atrial Fibrillation) clarify that “valvular AF”, which requires VKA treatment, specifically refers only to patients with mechanical heart valves or moderate to severe mitral stenosis.

As the most prevalent valvular condition in the senior population, aortic stenosis (AS) presents challenges for both diagnosis and treatment in these patients [24,25]. Furthermore, regardless of AF type, in high-risk patients with severe aortic stenosis undergoing TAVI, atrial fibrillation is associated with over twice the risk of cardiovascular and all-cause mortality. Therefore, in order to prevent an AF-related stroke in patients with severe AS and AF undergoing TAVI, anticoagulant usage is essential [26]. In patients with atrial fibrillation who are receiving TAVI, NOACs seem to have outcomes similar to VKAs, though more research is needed to validate these results [27]. The effectiveness of NOACs in individuals having mechanical valves in the aortic position remains uncertain, with the ongoing randomized PROACT-Xa trial designed to address these unknowns.

Although initially approved solely for the somewhat misleading term “non-valvular AF”, NOACs are now recognized as a viable option for a growing patient population with VHD. In this review, we explored the potential of NOAC treatment in individuals with AF and VHD, given their advantages such as the rapid onset and offset of anticoagulant effects, fixed dosing, fewer drug and dietary interactions, and no requirement for regular monitoring, as well as their safety-end efficacy profile [12].

## 2. NOAC in Native Valvular Disease

### 2.1. NOAC in Native Mitral Valvular Disease

**Figure 1:**
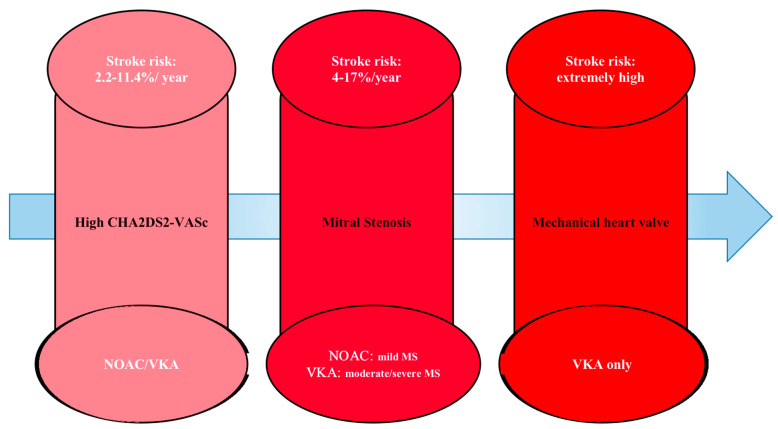
Anticoagulation therapy for patients with atrial fibrillation; NOAC: non-vitamin K antagonist oral anticoagulants; VKA: vitamin K antagonist; a CHA2DS2-VASc > 1 was defined as high; stroke risk was estimated according to Zhou et al. [29].

Thirty percent or more of AF patients also have some form of VHD. These patients face a higher risk of stroke and systemic thromboembolism compared to those with AF alone [28]. Although AF patients are generally more likely to have a stroke, there are three subgroups that are especially high-risk and require long-term anticoagulant therapy. Among these are patients with AF and mitral stenosis (MS), patients with AF and mechanical heart valves, and patients with non-valvular AF and high CHA2DS2-VASc scores (Figure 1).

Although the prevalence of rheumatic heart disease and mitral stenosis is low in developed countries, it affects a larger population in nations with low to moderate incomes, thus requiring an effective anticoagulant treatment [30]. In the REMEDY (Global Rheumatic Heart Disease) Registry, which included 3343 patients with rheumatic heart disease from 14 countries in Africa and Asia, the average age was 28 years, and 21% of these patients developed AF. Among those with AF, 77% received VKA treatment; however, 10% had not undergone an INR test within the 6 months prior to enrollment, and only 29% had an INR within the therapeutic range. These findings indicate that patients with mitral stenosis and AF from low- to moderate-income countries need alternatives to VKAs. Despite the inadequate treatment, only 2.4% of patients with a contraceptive indication had a stroke during a 2-year follow-up, likely due to their young age [31].

The 2023 ACC/AHA/ACCP/HRS Guideline for the Diagnosis and Management of Atrial Fibrillation recommends long-term anticoagulation with warfarin over NOACs in individuals with moderate or greater mitral stenosis and a history of AF, regardless of the CHA2DS2-VASc score, in order to avoid cardiovascular incidents such as stroke or death. For other patients with valve disease and AF, excluding those with moderate or severe mitral stenosis or a mechanical heart valve, NOACs are indicated over VKAs. Four large trials compared the effectiveness of VKA vs. NOAC as a therapy for patients with AF (Table 1). These include ARISTOTLE (Apixaban for Reduction in Stroke and Other Thromboembolic Events in Atrial Fibrillation), ROCKET-AF (Rivaroxaban Once Daily Oral Direct Factor Xa Inhibition Compared with Vitamin K Antagonism for Prevention of Stroke and Embolism Trial in Atrial Fibrillation), RE-LY (The Randomized Evaluation of Long-Term Anticoagulation Therapy), and ENGAGE AF (Effective Anticoagulation with Factor Xa Next Generation in Atrial Fibrillation).

In summary, 13,585 (19.0%) of the participants in these studies had a bioprosthesis or a previous valve intervention, or they had moderate to severe valvular illness. While mitral regurgitation accounted for most of these individuals (N = 10,633; 78.2%), a sizable portion of patients also had additional valve abnormalities or had undergone previous valve surgery [32,33,34,35]. Across all four trials, patients with VHD were found to be older and had a greater number of both cardiovascular and non-cardiovascular comorbidities compared to those without the condition. The primary outcomes of the trial, stroke and systemic embolism, as well as significant bleeding were more common in these types of patients. Nevertheless, the effect of NOACs compared to VKAs on the primary efficacy endpoint of stroke and systemic embolism was comparable for patients with and without valvular heart disease across all trials. Not a single trial revealed any evidence of heterogeneity in the interaction between different valve lesions and the effects of NOACs versus VKAs on key clinical outcomes. The European Society of Cardiology (ESC) and the American College of Cardiology/American Heart Association (ACC/AHA) guidelines state that NOACs are not inferior to VKAs in patients with AF and mitral regurgitation, based on the results of these subanalyses [36,37].

A retrospective observational study aimed to validate the efficacy of NOACs in patients with mitral stenosis and included 2230 patients (mean age 69.7 ± 10.5 years; 682 [30.6%] males). The study found that thromboembolic events occurred at a rate of 2.22% per year in the NOAC group, compared to 4.19% per year in the group treated with warfarin (aHR for NOAC: 0.28; 95% CI: 0.18 to 0.45). Intracranial hemorrhage rates were 0.49% in the group treated with NOAC and 0.93% in the group treated with warfarin (aHR for NOAC: 0.53; 95% CI: 0.22 to 1.26). These results indicate that the use of NOACs could be a suitable option for patients with mitral stenosis and atrial fibrillation. However, a significant drawback of the study was the failure to determine the severity of mitral stenosis and other valvular pathologies in a considerable proportion of patients [38].

The main issue remains if there is a possibility of NOAC treatment for moderate or severe mitral stenosis. Considering these factors, the ongoing DAVID-MS (DAbigatran for Stroke Prevention In Atrial Fibrillation in Moderate or Severe Mitral Stenosis) trial is designed to offer clinicians crucial details on the prevention of stroke strategies for patients with moderate or severe mitral stenosis and AF (Table 2). The trial aims to evaluate whether dabigatran is not inferior to VKA in preventing the primary outcome of stroke or systemic embolism [29]. The goal is to generate the required proof to establish international clinical practice guidelines for stroke prevention in patients with AF and mitral stenosis. There are numerous significant ramifications for the current study, especially for Asian nations. Asians are repeatedly shown to have a considerably elevated risk of cerebral hemorrhage than non-Asians in previous epidemiological research and subanalyses of the major NOAC trials, supporting the use of NOACs over warfarin treatment [39,40,41,42,43].

RISE MS was a small Iranian study that included 37 patients with moderate to severe mitral stenosis and AF within the previous 12 months who were randomized to receive either rivaroxaban 20 mg per day or warfarin. The study’s primary outcomes were symptomatic ischemic strokes and systemic embolic events observed over a year-long follow-up period. The findings suggested that NOACs are minimally as successful as VKAs in reducing the risk of thrombosis for AF patients with moderate to severe MS [44]. The principal limitation of this study is the small number of patients included.

Another significant ongoing study, INVICTUS (INVestIgation of rheumatiC AF Treatment Using VKAs, rivaroxaban, or aspirin Studies), is designed to ascertain the best anticoagulant method to avoid stroke in individuals with AF and valvular heart disease, particularly those with significant mitral stenosis [30]. The INVICTUS-VKA trial is an international, multicenter, investigator-initiated, randomized trial that tests the hypothesis that rivaroxaban 20 mg daily is similar (and potentially superior) to VKA (warfarin) in reducing the risk of stroke or systemic embolism in patients with rheumatic atrial fibrillation who are at high risk of stroke. Preliminary findings revealed that individuals with AF, rheumatic heart disease, and mitral stenosis possessed a mean duration to the main outcome event, such as stroke, systemic embolism, myocardial infarction, or death from vascular or unknown causes, of 1675 days with VKA treatment, in contrast to 1599 days with rivaroxaban (difference, –76 days [95% CI, –121 to –31]; *p* < 0.001). These results indicate that rivaroxaban is less effective than VKAs in preventing stroke, systemic embolism, myocardial infarction, or death. Furthermore, VKA treatment led to a significantly lower rate of composite cardiovascular events or mortality compared to rivaroxaban, without increasing the incidence of major bleeding, although the rate of fatal bleeding was not higher. These findings align with and support current guidelines. Until more information is available, the current European and American guidelines recommend VKA over NOAC in patients with AF and moderate to severe mitral stenosis.

### 2.2. NOAC in Native Aortic Valvular Disease

Clinical trials studying the administration of non-vitamin K antagonist oral anticoagulants in AF have not adequately represented patients who also have aortic stenosis [46]. The frequency of aortic stenosis in patients with AF is significant, ranging from 2% to 5%. Conversely, 20% to 30% of individuals with aortic stenosis additionally experience AF [7,47,48]. Individuals suffering from aortic stenosis particularly face the highest risk of stroke and bleeding among all valvular heart disease subtypes.

A well-known yet uncommon clinical feature of Heyde syndrome is gastrointestinal bleeding caused by angiodysplasias in patients with aortic stenosis (AS). Heyde syndrome is also characterized by acquired type IIA von Willebrand syndrome, which is brought on by blood passing through the stenotic aortic valve [49].

In randomized trials assessing the safety and effectiveness of NOACs vs. VKAs in individuals with AF, patients with AS were underrepresented, and their outcomes were variable [46]. Teppo et al. studied 5231 patients with AS and discovered that initial anticoagulant use was distributed as follows: warfarin in 73.3%, rivaroxaban in 9.6%, apixaban in 12.1%, dabigatran in 4.6%, and edoxaban in 0.4% of cases. The study demonstrated that these anticoagulants presented a comparable risk of death and bleeding to VKAs when drug exposures were assessed as time-varying variables and in intention-to-treat analyses from the initiation of NOACs. In the intention-to-treat analysis, initiating NOAC therapy was linked to a reduced risk of ischemic stroke compared to VKAs. In analyses considering time-varying OAC exposures, a similar but nonsignificant trend towards a diminished risk of ischemic stroke with NOACs was observed [46]. Patients with AS on warfarin and apixaban had similar bleeding and stroke rates according to the post hoc analysis of the ARISTOTLE trial [34]. Furthermore, AF and AS patients treated with NOACs had a decreased probability of significant bleeding but a higher risk of thromboembolism contrasted with VKAs, according to a recent Danish observational study [50].

In the ROCKET-AF trial, individuals with AS exhibited noticeably greater rates of severe bleeding, stroke, or systemic embolism compared to those without valvular heart disease (VHD). Further examination revealed that AS patients on a daily dose of 20 mg rivaroxaban had a substantially higher chance of serious bleeding but comparable rates of stroke or systemic embolism to those on warfarin. On the other hand, AS patients taking 5 mg of apixaban twice daily had similar rates of severe bleeding, stroke, or systemic embolism compared to those on warfarin. The ENGAGE AF-TIMI 48 study found that individuals with AS and AF exhibited an increased prevalence of stroke or systemic embolism than those without VHD. There have not been any direct comparisons between the effects of 60 mg edoxaban and warfarin in patients with AS or other VHD types [51].

No proof is currently available from the literature regarding a comparison between NOACs for aortic or mitral valve disease. The efficacy and safety of NOACs have been evaluated compared to vitamin K antagonists (VKA). Apixaban and rivaroxaban have not been directly compared and randomized in individuals with both AF and VHD. Dawwas et al. discovered that in a cohort of individuals with AF and VHD, compared to rivaroxaban, apixaban was associated with a lower incidence of cerebral or GI bleeding, ischemic stroke, and systemic embolism. The reported results may be partially explained by potential variations in pharmacokinetic or pharmacodynamic characteristics (rivaroxaban is supplied once a day, whereas apixaban is provided twice daily) [52].

## 3. NOAC in Mitral Valve Repair

Mitral valve repair is the favored treatment for asymptomatic patients with severe primary mitral regurgitation (MR) who have normal left ventricular systolic function (LVEF ≥ 60%) and a left ventricular end-systolic diameter (LVESD ≤ 40 mm), as well as for symptomatic individuals with significant primary MR resulting from rheumatic valve disorder. To prevent thromboembolic complications, the European Association of Cardio-Thoracic Surgery guidelines suggest oral anticoagulation with VKA for 3 months following surgery (class IIb recommendation, level of evidence C). Interestingly, the joint American Heart Association/American College of Cardiology guidelines on heart valve disease management do not provide a recommendation on this matter.

Transcatheter mitral valve repair (tMVR) is a newer treatment option that requires anticoagulation because certain prosthetic material may be thrombogenic. As physicians began integrating data from various studies and growing more familiar with NOACs, one study found that by the end of 2018, over 40% of patients who underwent either surgical mitral valve repair (sMVR) or tMVR were being treated with NOACs. The use of NOACs has steadily increased since 2014, although this rise did not lead to an overall increase in the use of anticoagulation therapy [53].

Despite the lack of randomized evidence, observational data indicate that treatment with acetylsalicylic acid (ASA) or VKAs after mitral valve replacement had a similar risk of thrombosis. VKAs are a more appropriate initial choice due to the high frequency of both new-onset and recurrent atrial fibrillation, the thrombogenic properties of the non-endothelialized repair components, and the relatively higher percentage of individuals who demonstrate resistance to aspirin (ASA) [37].

This retrospective cohort analysis of 1997 patients with AF undergoing either sMVR or tMVR revealed no significantly different results in the use of NOACs versus VKAs based on age, sex, race/ethnicity, median household income, comorbidities, prior stroke, or CHA2DS2-VASc score among those who received an anticoagulant. However, patients who underwent tMVR had a higher chance of receiving treatment with a NOAC compared to those who had sMVR (OR 2.09, 95% CI 1.31 to 3.34, *p* < 0.01) [53]. Surprisingly, over 40% of patients did not obtain anticoagulation during a 90-day period following release, and these rates remained stable throughout the study period, suggesting potential gaps in treatment knowledge. The study concluded that while NOAC use increased over time, with more than 40% of patients receiving a NOAC, this did not lead to a higher overall rate of anticoagulation.

## 4. NOAC in Bioprosthetic Mitral Valves

For patients without complications following bioprosthetic valve surgery, the risk of thromboembolism is highest for the first three months after the procedure; for individuals with AF, the risk is perpetual [54,55,56].

Since the release of the 2017 VHD Guidelines, the evidence favoring the use of NOACs over VKAs has grown significantly [36]. According to the most recent European guidelines, patients who have surgical bioprosthetic valves are recommended to take a single-agent oral anticoagulant as class I. Class IIa recommendations suggest that patients with AF and bioprosthetic valves consider NOAC three months after the surgical implantation of a bioprosthetic heart valve. According to ACC/AHA guidelines, patients who had a bioprosthetic valve installed more than three months ago (Class I) should get either NOAC or VKA; patients who had new-onset AF less than three months following bioprosthetic valve implantation (Class IIa) should receive VKA [37].

A portion of the data came from the RIVER (Rivaroxaban for Valvular Heart Disease and Atrial Fibrillation) trial, which evaluated the use of NOACs in individuals with bioprosthetic mitral valves that indicated lifetime anticoagulant use and atrial flutter or fibrillation [57]. The RIVER trial, conducted across 49 sites in Brazil, enrolled 1005 patients in a multicenter, open-label, randomized, non-inferiority study with a blinded evaluation of outcomes. Led by academic researchers, the trial involved individuals with a bioprosthetic mitral valve and AF, or flutter. Participants were randomly assigned to either dose-adjusted warfarin (target INR 2.0–3.0) or rivaroxaban 20 mg once daily (15 mg for those with creatinine clearance < 50 mL/min). The study followed these patients for a full year. A composite of severe bleeding, valve thrombosis, systemic embolism, stroke, hospitalization for heart failure, and significant bleeding was the main result. According to a net benefit endpoint at 12 months, the rivaroxaban group had lower rates of cardiovascular death (3.4% vs. 5.1%, HR 0.65, 95% CI 0.35–1.20), stroke (0.6% vs. 2.4%, HR 0.25, 95% CI 0.07–0.88), and major bleeding (1.4% vs. 2.6%, HR 0.54, 95% CI 0.21–1.35) than the VKA group. These findings confirm that rivaroxaban is not inferior to warfarin.

NOAC consistently provided benefits to all types of bioprosthetic valves. More than 200 participants who had successfully undergone surgical bioprosthetic valve implantation or repair to one or both of the aortic or mitral valves were enrolled in the ENAVLE trial [58]. For a duration of three months, patients were randomized to receive either warfarin (dosage adjustment to maintain INR 2.0–3.0) or edoxaban (60 mg once daily, or 30 mg in those with a CrCl 30–50 mL/min or with a body weight ≥ 60 kg). The primary efficacy outcome was death, clinical thromboembolic events, or silent intracardiac thrombosis. The main outcome rates were 3.67% for the warfarin group and 0% for the edoxaban group (*p* < 0.001 for noninferiority). When it came to preventing thromboembolism and major bleeding in the first three months following aortic or mitral surgical bioprosthetic valve implantation or repair, edoxaban was found to be non-inferior to warfarin (*p* = 0.013 for noninferiority). This was evident in the case of three patients (2.75%) in the edoxaban group and one patient (0.92%) in the warfarin group for intracranial hemorrhage.

In a 2021 study involving 2672 participants with bioprosthetic valve and AF, NOACs were compared to VKAs in terms of thromboembolic events and bleeding complications. The primary outcomes—ischemic stroke, systemic embolism, and transient ischemic attack—were found to be similar between the NOAC and VKA groups. However, NOACs significantly reduced the bleeding risk and also intracranial hemorrhage and gastrointestinal bleeding. The two groups had similar rates of all-cause death [59].

In a post hoc analysis from the ENGAGE AF-TIMI 48 trial, edoxaban revealed a comparable rate of stroke/systemic embolism (HR, 0.37; *p* = 0.15) and major bleeding (HR, 0.5; *p* = 0.26) compared to warfarin. The analysis included 191 AF patients with aortic (31.4%) or mitral (68.6%) bioprosthetic valve replacements and found that edoxaban was associated with a reduced incidence of myocardial infarction, stroke, or cardiovascular death (HR, 0.36; *p* = 0.03) compared to warfarin [60]. Furthermore, in the ARISTOTLE trial, 156 patients with AF, a bioprosthetic valve, or valve surgery showed no appreciable differences in safety or efficacy results between apixaban and warfarin [61]. All these studies support the guidelines recommendation of NOAC treatment three months after the surgical implantation of a bioprosthetic heart valve.

## 5. NOAC in Mechanical Mitral Prosthetic Valves

Treatment for patients with severe VHD mostly consists of replacing the damaged native valve with a prosthetic valve [54,62,63].

Individuals with severe VHD tend to have longer survival times and improved quality of life with prosthetic valves; however, there remains a potential risk of thrombotic events [55,56]. The number of people receiving surgical therapy for mitral stenosis is increasing; by 2009, over 4 million people had prosthetic valves installed, with 300,000 of those procedures occurring annually. However, not enough information is available to determine the safety and efficacy of NOACs in these individuals [64]. Regardless of any medical conditions, people in sinus rhythm with artificial heart valves should take oral anticoagulants for the rest of their lives due to the danger of thrombotic events.

In the 2021 ACC/AHA Guideline for the Management of Patients With Valvular Heart Disease, the class 1 recommendation is to treat patients with a mechanical prosthetic valve with anticoagulation with a VKA. The 2023 ACC/AHA/ACCP/HRS Guideline for the Diagnosis and Management of Atrial Fibrillation further supports that for patients with mechanical heart valves, whether or not they have AF, long-term anticoagulation should be managed with VKAs to avoid valve thrombosis, as NOACs are not recommended for this purpose.

For individuals with moderate to severe mitral stenosis with AF, there have been no completed randomized controlled studies for NOACs [65]. The explanation is that patients with this type of VHD were excluded from studies comparing VKAs and NOACs due to their increased risk of thromboembolism [66]. Regarding the use of NOACs in patients with mechanical heart valves, the findings of the RE-ALIGN study are evidential, illustrated by an elevated number of bleeding and ischemic events in the NOAC arm. NOAC are not safe for the first few days following surgery due to mechanical valves’ evolving thrombogenicity over time. Another reason is because surgery-induced trauma triggers a brief hypercoagulable state, triggering the extrinsic coagulation pathway, which VKAs block but dabigatran does not [65]. Prolonged activation of the coagulation cascade by mechanical heart valves results in elevated local thrombin concentrations. There are required higher doses of dabigatran to inhibit thrombin, doses that are dangerous [67]. Furthermore, 405 individuals with mechanical prosthetic bileaflet heart valves in the aortic or mitral position receiving dabigatran or VKA treatment were examined in the RE-ALIGN study (Randomized, Phase II Study to Assess the Safety and Pharmacokinetics of Oral Dabigatran Etexilate in Patients after Heart Valve Replacement) [23]. As a result of an abundance of ischemic and bleeding events in the dabigatran arm, the study was terminated after 252 patients were enrolled. This demonstrated that VKA was more efficacious than dabigatran in preventing stroke and systemic embolism in individuals with mechanical prosthetic valves. There are multiple issues regarding these results. The majority of participants in RE-ALIGN were enlisted early and post-operatively, and the worst adverse events emerged in this cohort of patients treated with dabigatran. Major bleeding and stroke occurred in the first 90 days after surgery, suggesting that dabigatran may not be effective early after surgery. On the other hand, mechanical valve thrombogenicity varies with time. Initially, parts of a prosthetic valve are highly thrombogenic, but they gradually develop a less thrombogenic neointimal covering [68]. Additionally, there is a transient hypercoagulable state following surgery, which activates the extrinsic coagulation pathway—a process more effectively inhibited by VKAs than by dabigatran. In this trial, dabigatran’s dose was adjusted to as high as 300 mg twice a day, which is double the recommended dosage for those with non-valvular AF and differs from its standard use in clinical practice and other trials. Moreover, dabigatran’s mechanism of action, as a direct thrombin inhibitor, may have been a suboptimal choice. Dabigatran inhibits thrombin, the final step in the coagulation cascade, on a 1:1 basis, while mechanical heart valves continuously trigger the coagulation cascade, leading to very high local thrombin levels. The increased anticoagulant effectiveness of VKAs is partially attributed to upstream inhibition as well as their inhibition of several components in the intrinsic and extrinsic coagulation pathways [65]. It is plausible to suggest that Xa inhibitors, by targeting a point earlier in the coagulation pathway, may more effectively inhibit thrombin generation in mechanical heart valves. Furthermore, the RE-ALIGN trial included any mechanical bileaflet valve in either the mitral or aortic position, with the understanding that mechanical valves in the mitral position are known to be more thrombogenic than those in other positions.

In this study, rivaroxaban was assessed in patients who had undergone isolated mitral valve replacement with a mechanical prosthesis and experienced unstable INR control at least three months post-surgical therapy [69]. The patients received rivaroxaban 15 mg twice per day for 90 days. Throughout the three-month follow-up, there were no cases of intracardiac thrombus, ischemic or hemorrhagic stroke, reversible ischemic neurological deficit, hospitalization, or death. Additionally, spontaneous echocontrast resolved in two patients. The primary research constraint was the limited number of individuals and the suspension of the study even with a high patient satisfaction rating and a wish to keep using the trial medication.

Current guidelines suggest an INR target range of 2.5 to 3.5 for individuals with a mechanical mitral prosthesis. The On-X bileaflet mechanical valve is engineered to optimize hemodynamic flow, reduce pannus formation, and minimize thrombus risk, offering a low thrombotic profile. The PROACT (Prospective Randomized On-X Anticoagulation Clinical Trial) Mitral trial was a randomized controlled noninferiority study that explored the safety and effectiveness of using lower doses of warfarin than typically recommended for patients with an On-X mechanical mitral valve. Beyond a typical follow-up of 4.1 years, 401 patients were assigned either to a low-dose warfarin group (target INR, 2.0–2.5) or a standard-dose warfarin group (target INR, 2.5–3.5). The trial found that low-dose warfarin did not meet the criteria for noninferiority compared to standard-dose warfarin in terms of the composite primary outcome, which included thromboembolism, valve thrombosis, and bleeding events [70].

In conclusion, due to the lack of valid studies, ESC guidelines recommend chronic VKA use regulated by INR levels for mechanical mitral prosthetic valves; NOACs currently have no beneficial proven role in these patients. The question that remains and needs further investigation is if NOACs can be used in mechanical mitral valves late after intervention, after a certain number of months with VKA treatment.

## 6. NOAC in Transcatheter Aortic Valve Implantation

Current guidelines strongly recommend lifelong oral anticoagulation therapy for patients who have undergone transcatheter aortic valve implantation (TAVI) and have other existing indications for OAC.

TAVI has been widely studied as a treatment for individuals with severe, symptomatic AS, with numerous randomized controlled trials conducted across different levels of surgical risk [71,72]. TAVI patients are typically advanced in age and often suffer from various comorbidities, along with a range of unique and clinically significant risk factors. After TAVI, about half of the patients require both antiplatelet and oral anticoagulation therapy, though the specific combination and duration of these treatments can vary considerably [73].

For patients with nonvalvular AF, NOACs have shown numerous benefits. Given that over 40% of patients undergoing TAVI have AF and a heightened risk of stroke, it is still uncertain if NOACs can be effectively used in TAVI patients and whether their efficacy and safety profiles are comparable to those of VKAs in this population [74]. After TAVI, the ideal antithrombotic strategy should be chosen by carefully balancing the risks of bleeding and ischemia. This decision should also take into account any concurrent conditions, such as coronary artery disease or AF, which might affect the choice between antiplatelet and anticoagulant therapy [75].

In a retrospective observational analysis, Geis et al. assessed the 6-month outcomes of individuals with TAVI and AF receiving NOAC (N = 154) and VKA (N = 172), with no statistically significant changes in terms of severe bleeding, embolism, death, or stroke [76]. Using a single-center observational analysis, Seeger et al. examined the results of apixaban (n = 141) and VKA (n = 131) at 1 year following TAVI. The results showed no notable variations in the overall death rate, hemorrhage, or stroke between the two groups [77]. A total of 8962 TAVI patients treated with VKAs and 2180 TAVI patients treated with NOACs were included in the French registries (FRANCE-TAVI and FRANCE 2), which demonstrated a higher 3-year risk of mortality and serious bleeding for those on conventional anticoagulants [78]. Conversely, in a Danish registry involving 219 participants treated with NOACs and 516 patients treated with VKAs, the 3-year risks of thromboembolic events, hemorrhage, and all-cause mortality were discovered to be similar in the two groups [79].

GALILEO40, a recent randomized trial, compared dual-antithrombotic therapy with the randomization of patients receiving either Rivaroxaban 10 mg or Aspirin (ASA) 75–100 mg after TAVI. The results showed that the Rivaroxaban + ASA group experienced greater rates of bleeding and thrombosis. Another randomized trial, POPular, reported decreased bleeding rates in the oral anticoagulation-only group when patients were randomized after TAVI to receive oral anticoagulation only (NOAC or Warfarin) vs. oral anticoagulation with clopidogrel [51]. An observational study indicated that, after adjusting for potential confounders, the risk of ischemic events at one year is higher with NOACs compared to VKAs [80].

**Figure 2:**
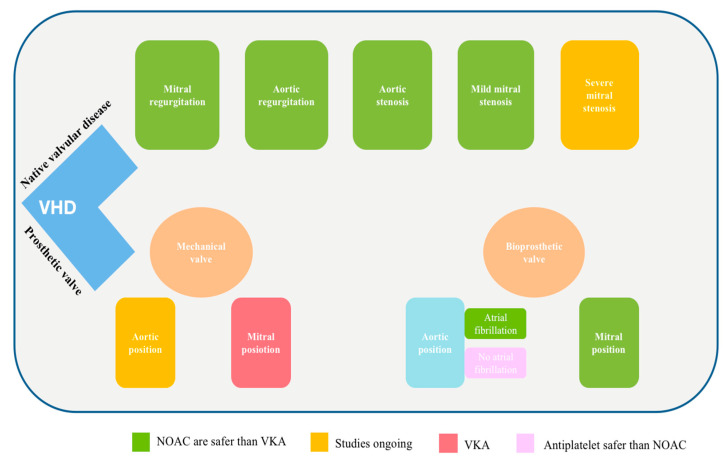
Current recommendation for NOAC in valvular heart disease; VHD: valvular heart disease; NOAC: non-vitamin K antagonist oral anticoagulants; VKA: vitamin K antagonist.

Recent research comparing the safety and efficacy of direct oral anticoagulants (NOACs) to standard care in individuals without indications for oral anticoagulants (OACs) after TAVI has found that NOACs are connected to a higher rate of all-cause mortality. However, NOAC treatment was shown to significantly reduce the incidence of reduced leaflet motion (RLM) (RR: 0.19, 95% CI [0.09, 0.41], *p* = 0.0001) and hypoattenuated leaflet thickening (HALT) (RR: 0.50, 95% CI [0.36, 0.70], *p* = 0.0001) [50] (Figure 2).

According to American and European standards, patients with bioprosthetic heart valves may consider NOACs, unlike mechanical heart valves, but there are certain important limitations. Patients with a bioprosthetic heart valve or transcatheter valve implantation (TAVI) may only require a single lifelong antiplatelet medication for long-term antithrombotic therapy, unless they have other indications or comorbidities [36,37,81]. For patients already on anticoagulation therapy before undergoing TAVI, the recommendation is to continue anticoagulation with either warfarin or a NOAC without adding any antithrombotic medications, as long as there has been no recent coronary stenting (e.g., within the last 3 months) [82].

Edoxaban or warfarin was administered to individuals with pre-existing AF in the ENVISAGE-TAVI AF study 1426 within 12 h to 7 days following successful TAVI. For the primary efficacy endpoint, edoxaban was found to be noninferior to warfarin at a median follow-up period of 18 months (HR 1.05, 95% CI 0.85–1.31, noninferiority margin 1.38; *p* = 0.01 for noninferiority). Additionally, the edoxaban group experienced a higher primary safety outcome of significant bleeding (HR 1.4, 95% CI 1.03–1.91, noninferiority margin 1.38; *p* = 0.93 for noninferiority) [83]. When it comes to selecting an anticoagulant, safety data on apixaban are available for patients undergoing TAVI who already possess proof of anticoagulation. Younger patients with surgically implanted bioprosthetic valves who are at least three months post-procedure may be considered for rivaroxaban use [84,85,86]. However, evidence is still deficient in terms of using NOACs in AF patients within the first three months after surgical or transcatheter bioprosthesis implantation. Additionally, observational information on NOAC use following transcatheter aortic valve implantation remains inconsistent.

## 7. NOAC and Mechanical Aortic Valve

Anticoagulation is required for mechanical heart valves to avoid thrombosis, with warfarin being the only approved medication for this purpose. The target international normalized ratio (INR) varies based on the type and location of the valve, as well as associated thrombosis risk factors. Non-vitamin K oral anticoagulants are not accepted for use in individuals with artificial heart valves, even if they have other indications such as AF or venous thromboembolism [82].

Considering the RE-ALIGN study, individuals with mechanical heart valves have been excluded from major NOAC trials from their inception [23].

In this study, 252 participants with mechanical mitral or aortic valves were randomized at a 2:1 ratio to receive either warfarin or dabigatran. The dabigatran group experienced an increased risk of bleeding and stroke, which prompted the study’s early discontinuation. Consequently, dabigatran is contraindicated for patients with mechanical heart valves because of the heightened risk of bleeding and stroke. Since this trial, few studies have explored the use of NOACs in patients with mechanical heart valves. Although apixaban, edoxaban, and rivaroxaban carry warnings for this patient population, they are not explicitly contraindicated [82].

The On-X mechanical aortic prosthesis, due to its unique design, may possess a decreased risk of thrombosis compared to conventional mechanical valves, allowing for the use of a reduced dosage of warfarin. In a trial conducted at least three months after surgery, patients with this valve were treated with either standard-dose warfarin (target INR range, 2.0–3.0) or apixaban (5 mg twice per day). Throughout the study, the majority of participants (84%) continued taking low-dose aspirin. However, after enrolling 863 individuals (mean age, 56 years; 24% women), the trial was abruptly terminated because of an increased frequency of thromboembolic events in patients receiving apixaban [86].

## 8. NOAC in Patients with VHD and Other Comorbidities

### 8.1. Acute Thrombosis

The finding that stroke rates were 10.2% among AF patients receiving NOAC anticoagulation emphasizes the necessity of carefully examining the prescription appropriateness and anticoagulation adherence in this patient population [87]. Thrombolysis is advised for patients presenting with acute ischemic stroke within 4.5 h of the beginning of symptoms to achieve a better result. However, it cannot be given to individuals whose INR in VKAs is greater than 1.7 or within 24 h after the last NOAC dosage. An evaluation of the NOACs’ plasma concentration may be helpful for up to four hours after the last medication intake; if the concentration of NOACs is less than 30 ng/mL, thrombolysis may be recommended [88].

Regarding individuals with acute coronary syndrome and AF, when taking NOAC, it is recommended to include a P2Y12 inhibitor and a low-dose aspirin (150–300 mg), particularly in cases with ST-elevation myocardial infarction (STEMI). For elective procedures or non-ST elevation myocardial infarction (NSTEMI), when a catheter operation is performed at least 12 to 24 h after the last NOAC dose, bridging therapy with LMWH (fondaparinux or enoxaparin) should be administered. In these situations, NOACs should also be temporarily discontinued [89].

To avoid early stent thrombosis, triple antithrombotic therapy (TAT) with OAC (NOAC or VKA) and two antiplatelet medications (aspirin and a P2Y12 inhibitor) is required with an individualized duration according to bleeding and ischemic risks [90].

### 8.2. Coronary Heart Disease

As the recent guidelines suggest, the management of patients with VHD, AF, and associated chronic stable coronary heart disease (CHD) demands a cautious balancing act between anticoagulation for stroke prevention, antiplatelet therapy for coronary artery disease, and minimizing the risk of bleeding [12,91].

Traditionally, patients with VHD (especially those with mechanical heart valves or moderate to severe mitral stenosis) and AF are managed with warfarin due to its efficacy in reducing the risk of thromboembolic events. For patients with VHD (excluding those with mechanical heart valves or significant mitral stenosis) and AF, NOACs can be considered, although caution is needed when CHD is present. For stable CHD, aspirin (75–100 mg daily) is typically recommended as an antiplatelet agent to reduce the risk of myocardial infarction and other coronary events [12]. If the patient has stable coronary artery disease without recent stenting or acute coronary syndrome, monotherapy with an anticoagulant (warfarin or a NOAC) may be sufficient, and aspirin may be added based on individual risk assessments. For patients with stable coronary heart disease (CHD) and a high risk of thromboembolic events, anticoagulation therapy (warfarin or NOAC) combined with low-dose aspirin may be utilized. However, this combination should be used cautiously due to the increased risk of bleeding. For some individuals, particularly those at high bleeding risk, it may be appropriate to discontinue aspirin and rely solely on anticoagulation [91].

Ongoing assessment of bleeding risks versus thromboembolic risks is critical, with adjustments to therapy as needed based on patient-specific factors and any changes in clinical status. Furthermore, lifestyle and risk factor management is crucial, such as the management of hypertension, diabetes, and hyperlipidemia to lessen overall cardiovascular risk and encourage a diet low in fat, frequent exercise, and quitting smoking to further reduce cardiovascular risk.

Regarding the use of antiplatelets in patients with only AF and VHD, the AVERROES (Apixaban Versus Acetylsalicylic Acid [ASA] to Prevent Stroke in Atrial Fibrillation Patients Who Have Failed or Are Unsuitable for Vitamin K Antagonist Treatment) trial is notable for being the only study that has directly compared a NOAC, apixaban, with aspirin in individuals with AF. Patients with AF who were not eligible for VKA treatment were evaluated on aspirin and apixaban 5 mg twice a day in this study. The trial was halted early due to the clear benefits observed because apixaban, as opposed to aspirin, dramatically lowered the risk of stroke or systemic embolism (HR, 0.45; 95% CI, 0.32–0.62; *p* < 0.001). Importantly, the two groups’ major bleeding did not differ significantly from one another (HR, 1.13; 95% CI, 0.74–1.75; *p* = 0.57) [92].

In light of these results, antiplatelet treatment alone is advised (whether as monotherapy or aspirin combined with clopidogrel) and should not be used for stroke prevention in patients with AF, regardless of their stroke risk. This underscores the importance of anticoagulation over antiplatelet therapy in this patient population for effective stroke prevention [91].

## 9. Conclusions

The presence of aortic stenosis, aortic regurgitation, mitral regurgitation, and mild mitral stenosis can be treated with NOACs over VKA in patients with AF, mirroring their usage in individuals without VHD. Consensus guidelines advocate for the use of NOACs instead of VKAs in these particular patients. For individuals with moderate or severe mitral stenosis, there are a lack of clinical trials evaluating the risk and performance of NOACs to VKAs, although a significant study implies potential benefits, and two trials are currently in progress. In contrast, for individuals with mechanical mitral valves, dabigatran has been demonstrated to raise the risk of thromboembolic events and major bleeding compared to VKAs, leading to the contraindication of NOACs in this group. The effectiveness and safety of other NOACs in these individuals, as well as the effectiveness of NOACs in those with mechanical valves in the aortic position, remain uncertain. The ongoing randomized PROACT-Xa trial is expected to provide insights into these issues. The pivotal trials also encompassed patients with AF and surgical bioprosthetic valves, with subsequent validation of the safety and effectiveness of rivaroxaban in the RIVER trial. In patients undergoing transcatheter aortic valve implantation (TAVI) who do not have atrial fibrillation or other reasons for anticoagulation, NOACs lower the chance of subclinical leaflet thrombosis but also raise the risk of major bleeding and thromboembolic complications compared to antiplatelet therapy, making them less favorable in these situations. For individuals with AF undergoing TAVI, outcomes with NOACs show as comparable to VKAs, although additional data are warranted.

Although NOACs were initially approved only for “non-valvular AF”, they are now recognized as an effective course of therapy for an increasing number of patients with valvular heart disease.

## Figures and Tables

**Table 1:** Main trials involving patients with atrial fibrillation and valvular heart disease. NOAC vs. VKA in major trials enrolling patients with valvular heart disease; VKA: vitamin K antagonist; TAVI: transcatheter aortic valve implantation; AS: aortic stenosis; MR: mitral regurgitation; AR: aortic regurgitation; TR: tricuspid regurgitation.

Study	Comparison	Major Outcomes	CHA2DS2-VASc Scores	Age	Comorbidities	Percent of Patients with Particularly Aetiology of Valvopathy	Reference
ARISTOTLE	Apixaban vs. VKA	When it came to avoiding stroke or systemic embolism, apixaban outperformed warfarin, produced less bleeding, and decreased mortality.	the mean CHADS2 score was 2.1	median age was 70 years	19% previously experienced a transient ischaemic attack or stroke, or systemic embolis	19.4% Mitral regurgitation 0.7% Mitral stenosis (mild) 2.1% Aortic stenosis	[10]
ROCKET-AF	Rivaroxaban vs. VKA	Warfarin and rivaroxaban emerged to b[e equally efficient in averting strokes and systemic embolism.	Mean score 3–3.5	Median age 73	90.5% with hypertension, 62.5% with heart failure, and 40.0% with diabetes. A history of stroke, systemic embolism, or transient ischaemic attack was present in 54.8% of the patients.	12.4% mitral regurgitation 3.4% aortic regurgitation 1.5% aortic stenosis	[9]
RE-LY	Dabigatran vs. VKA	Compared to warfarin, dabigatran 150 mg was linked to equal rates of serious bleeding but lower incidence of stroke and systemic embolism. Dabigatran 110 mg showed comparable statistics for systemic embolism and stroke, along with decreased rates of severe bleeding.	mean CHADS2 score was 2.1	65 to 74 years	diabetes mellitus, hypertension, or coronary artery disease	17.1% Mitral regurgitation 1.1% Mitral stenosis (mild) 2.6% Aortic stenosis	[8]
ENGAGE AF	Edoxaban vs. VKA	When it came to preventing stroke or systemic embolism, both once-daily edoxaban regimens were comparable to warfarin.	Mean score 2.8	Median age 72	Diabetes mellitus, hypertension, congestive heart failure, prior stroke	10.7% mitral regurgitation 1.7% aortic regurgitation 0.8% aortic stenosis	[11]

**Table 2:** Key studies comparing NOACs and VKAs for mitral stenosis.

Study	Population	Comparison	Major Outcomes	CHA2DS2-VASc Scores	Age	Comorbidities	Percent of Patients with Particularly Aetiology of Valvopath	Reference
DAVID-MS	Moderate or severe rheumatic mitral stenosis	Dabigatran vs.VKA	not yet conducted	-	>18 years	moderate or severe mitral stenosis	on-going	[29]
RISE MS	Atrial fibrillation with mitral stenosis	Rivaroxaban vs. warfarin	Over the course of a one-year follow-up, there were no differences in ischaemic stroke, systemic embolic events, or severe bleeding between the rivaroxaban and warfarin groups.	was not defined	Mean age 56–60	Hypertension, diabetes, heart failure, ischemic cerebrovascular accidents, coronary artery disease	All patients included had moderate to severe mitral stenosis	[44]
INVICTUS-VK	atrial fibrillation and moderate or severe rheumatic mitral stenosis	Rivaroxaban vs.VKA	Compared to rivaroxaban medication, vitamin K antagonist therapy resulted in a decreased composite rate of cardiovascular events or mortality without a greater bleeding rate.	CHA2DS2VASc score of at least 2	average age of the patients was 50.5 years	Rheumatic heart disease and atrial fibrillation, hypertension, coronary artery disease, diabetes mellitus, congestive heart failure	Moderate-to-severe mitral stenosis was present in 81.9% of the patients	[45]

## Data Availability

No new data were created or analyzed in this study. Data sharing is not applicable to this article.
